# Effect of music therapy on stress in chemically dependent people: a quasi-experimental study

**DOI:** 10.1590/1518-8345.2456.3115

**Published:** 2019-01-14

**Authors:** Gunnar Glauco De Cunto Taets, Rafael Tavares Jomar, Angela Maria Mendes Abreu, Marcia Alves Marques Capella

**Affiliations:** 1 Universidade Federal do Rio de Janeiro, Departamento de Fundamentos do Cuidado de Enfermagem, Macaé, RJ, Brazil.; 2 Instituto Nacional de Câncer José Alencar Gomes da Silva, Coordenação de Assistência, Rio de Janeiro, RJ, Brazil.; 3 Universidade Federal do Rio de Janeiro, Escola de Enfermagem Anna Nery, Rio de Janeiro, RJ, Brazil.; 4 Universidade Federal do Rio de Janeiro, Instituto de Biofísica Carlos Chagas Filho, Rio de Janeiro, RJ, Brazil.

**Keywords:** Music Therapy, Related Disorders Substance Use, Dependency, Emotional Stress, Drug Addition, Public Health, Musicoterapia, Transtornos Relacionados ao Uso de Substâncias, Dependência, Estresse Emocional, Adição a Drogas, Saúde Pública, Musicoterapia, Transtornos Relacionados con el Uso de Sustancias, Dependencia, Estrés Emocional, Adición de Drogas, Salud Pública

## Abstract

**Objective:**

to evaluate the effect of music therapy on the stress of chemically dependent people.

**Method:**

quasi-experimental study conducted at a philanthropic institution with 18 chemically dependent people undergoing treatment. Salivary cortisol (stress hormone) was collected in three moments: before, 60 minutes after, and 120 minutes after a music therapy group intervention. Statistical analysis adopted a significance level of *p* < 0.05 and used the Wilcoxon and Kruskal-Wallis non-parametric tests.

**Results:**

after 60 minutes of intervention, there was a statistically significant reduction in mean salivary cortisol levels (*p* < 0.001). A reduction was also noted after 120 minutes, but without statistical significance (*p* = 0.139).

**Conclusion:**

a single session of 60 minutes of group music therapy was able to reduce stress (salivary cortisol levels) of chemically dependent people.

## RESUMO

**Objetivo:**

avaliar o efeito da musicoterapia sobre o estresse de dependentes químicos.

**Método:**

estudo quase-experimental realizado em instituição filantrópica com 18 dependentes químicos em tratamento. O cortisol salivar (hormônio do estresse) foi coletado antes, 60 e 120 minutos após única intervenção musicoterapêutica realizada em grupo. A análise estatística adotou nível de significância de *p* < 0,05 mediante aplicação dos testes não paramétricos de Wilcoxon e de Kruskal-Wallis.

**Resultados:**

após 60 minutos da intervenção musicoterapêutica, houve redução estatisticamente significante nas médias dos níveis de cortisol salivar (*p* < 0,001). Após 120 minutos, também houve redução, mas sem significância estatística (*p* = 0,139).

**Conclusão:**

sessão única de 60 minutos de musicoterapia em grupo mostrou-se capaz de reduzir o estresse (níveis de cortisol salivar) de dependentes químicos.

## RESUMEN

**Objetivo:**

evaluar el efecto de la musicoterapia sobre el estrés de dependientes químicos.

**Método:**

estudio cuasi-experimental realizado en institución filantrópica con 18 dependientes químicos en tratamiento. El cortisol salivar (hormona del estrés) fue recogido antes, 60 y 120 minutos después de una única intervención musicoterapéutica realizada en grupo. El análisis estadístico adoptó un nivel de significancia de p <0,05 mediante la aplicación de las pruebas no paramétricas de Wilcoxon y de Kruskal-Wallis.

**Resultados:**

después de 60 minutos de la intervención musicoterapéutica, hubo reducción estadísticamente significativa en las medias de los niveles de cortisol salivar (*p* <0,001). Después de 120 minutos, también hubo reducción, pero sin significancia estadística (*p* = 0,139).

**Conclusión:**

uma única sesión de 60 minutos de musicoterapia en grupo se mostró capaz de reducir el estrés (niveles de cortisol salivar) de dependientes químicos.

## Introduction 

Drug use is as old as mankind. First used as a means to connect with deities and then as an escape from reality or as facilitators of creativity and expression, drugs can bring serious problems to humans, affecting physical, psychological, social and spiritual dimensions^1^. 

Today, drug use has reached alarming proportions throughout the world, and has been associated with violence and organized crime, reaching people of all walks of life and at increasingly earlier ages. The banalization of consumption and excessive publicity of licit drugs such as alcohol and tobacco contribute to increase the abusive use and addiction to chemical substances^2^.

According to the fifth edition of the Diagnostic and Statistical Manual of Mental Disorders of the American Psychiatric Association (DSM-5), the term harmful use of drugs characterizes a type of use that results in physical or mental harm, and the term drug abuse refers to the social consequences of problematic use, provided the typical phenomena of dependence, such as compulsivity, tolerance and abstinence, are absent^3^.

Drug dependence is a worldwide phenomenon that brings several consequences for the addicted individuals and for those who live with them, whether in the physical, psychic or social spheres. In the physical field, it causes diseases that can lead to death; in the psychic, it may cause psychological dependence; and in the social, it brings problems in family relationships, at work, and with the judicial system^4^.

A key feature of chemical dependence is the presence of psychophysiological symptoms that indicate that the individual continues to use a drug despite of significant problems related to it. There is a pattern of repeated self-management that generally results in tolerance, abstinence, and compulsive substance use^3^. 

Stress is considered to be the factor that most contributes to compulsive behavior during the course of drug dependence^5^. There are hypotheses that dependence is implied in motivational mechanisms, and that the motivational state is controlled by basic processes of homeostatic regulation^5^.

A systematic review on the association between stressors and relapse in users of psychoactive substances found that chronic and acute stress events significantly increase the risk of relapse, with acute events increasing by almost three-fold the risk of relapses and decreasing the time between them, and found that therapeutic intervention is necessary in the treatment of the most vulnerable chemically dependent patients^6^.

Hormonal responses to stress include increased *growth hormone* (GH) secretion, activation of immune system cells such as monocytes, neutrophils, lymphocytes and *Natural Killer* cells, increased interleukins, increased secretion of thyroid stimulating hormone (TSH), increased parathyroid secretion (PTH), increased vasopressin and corticotrophin-releasing factor^7^. 

Cortisol, the main glucocorticoid released by the adrenal cortex under stressful situations, is known as the stress hormone; its production and secretion raises during and after exposure to stressors^8^. Thus, salivary cortisol is an important indicator of stress, and an effective, accessible, fast and non-invasive measure of this phenomenon. 

Music therapy has been consolidated as a co-adjuvant in the treatment and care for users of psychoactive substances in public institutions, specialized clinics and therapeutic communities^1^. Within a treatment program, music therapy can involve both sonorous-musical resources, and also expression and movement. Musical sounds, integrated in the system of representations that gives it specific power, surprises not only because it intervenes directly in the individual’s state of consciousness, but also because of its ability to influence people’s behavior collectively^9^.

The question or problem that this study intended to answer was the following: Is music therapy able to reduce stress in chemically dependent people? The primary purpose of music therapy is to facilitate the opening of communication channels and/or the rehabilitation of physical, emotional, mental, social and cognitive needs^9^. Therefore, the investigation whether the use of music therapy contributes to the treatment and rehabilitation of chemically dependent people deserves attention, since music therapy has the goal of enhancing the potential and/or reestablishing the functions of individuals so that they may achieve a better intra and/or interpersonal integration and, consequently, a better quality of life by means of prevention, rehabilitation or treatment^1^. 

Considering the hypothesis that music therapy can reduce stress, the objective of this study was to evaluate the effect of music therapy on the stress of chemically dependent people.

## Method

This was a quasi-experimental before/after study conducted at a philanthropic institution located in the city of Rio de Janeiro, Brazil, which offers multiprofessional care (psychiatry, psychology and music therapy) to chemically dependent people and their relatives/friends. 

The target population of this research consisted of the 28 chemically dependent people undergoing psychiatric and psychological treatment who participated in a single session of group music therapy conducted in December 2016 at the institution where the study was performed. Out of the twenty-five dependents, five declined to participate, and out of the 23 who accepted, 18 were included in the intentional sample of this work because they met the following eligibility criteria: abstinence from drug use (licit or illicit); 18 years of age or over; non-use of cortisol inhibitors such as glutamine, vitamin C, whey protein, green tea, magnesium, prednisone, and dexamethasone; and not affected by Addison’s disease or Cushing’s syndrome. For the evaluation of these eligibility criteria, the chemically dependent patients were interviewed by trained nurses in a reserved place without the presence of third parties.

Music therapy in this study can be defined as the use of music and/or its elements (sound, rhythm, melody and harmony) by a qualified music therapist, with a client or group, in a process that facilitates and promotes communication, relationship, learning, mobilization, expression, organization and other relevant objectives, in order to achieve physical, emotional, mental, social and cognitive needs^1^. 

The single session of group music therapy took place between 18:00 and 20:00 hours and lasted 120 minutes. It was conducted by a music therapist who, accompanied by a guitarist, sang with the purpose of encouraging the addicts to do the same, who had the lyrics of the songs printed in hands. The songs used were chosen by the participants themselves and composed a repertoire of 13 popular Brazilian songs by several singers: Beto Guedes, Cazuza, Elis Regina, Lenin, Pixinguinha, Raul Seixas, Renato Russo, Roberto Carlos, Sandra de Sá and Tim Maia. It should be emphasized that the choice for a single intervention is due to the fact that this study intends to evaluate the immediate effect of music therapy on stress. 

In the sound-musical area, specifically in music therapy, techniques of recreation and vocal improvisation are used, as in the present study. In these occasions, the participant learns, executes, transforms and interprets any piece of music or a complete song. The receptive technique was also used in addition to these methods; in this case, the participant listens to the song in execution and responds to the experience in silence or verbally^10^. 

Cortisol was used as biomarker in the present study as a resource for diagnosis of stress because it is considered the *stress hormone* in the literature^11^ ^-^ ^12^. To measure the level of this biomarker, properly trained nurses collected saliva from the participants in three moments: before the music therapy setting, and 60 minutes and 120 minutes after starting, using a cotton swab (Salivette^®^) held for one to two minutes under the tongue. Then the swab, identified with the number assigned to each participant, was stored in a thermal box to be taken to the laboratory where it was properly stored for further analysis of cortisol level^12^. The content of cortisol in the saliva samples was measured by electrochemiluminescence immunoassay. The cortisol reference value adopted by the laboratory that analyzed the saliva samples was < 0.252 ug/dL, which corresponds to the values normally found from 4:00 p.m. to 8:00 p.m., which was the time interval when the samples were collected.

Additionally to saliva samples for cortisol analysis, properly trained nurses collected sociodemographic and clinical data of the patients, namely: age, sex, self-reported skin color, schooling, and drug(s) to which the patient was addicted. 

Data were initially submitted to the exploratory statistical technique, and simple frequency distribution was used to describe the studied population (sex, age and drug addiction). Afterwards, salivary cortisol levels before, 60 minutes after, and 120 minutes after the music therapy session were compared through the Wilcoxon and Kruskal-Wallis non-parametric tests appropriate to compare, respectively, two and three or more groups. The analyses were carried out in the Graph Pad Prism 7 software and the level of significance adopted was *p* < 0.05.

The present study was approved by the Research Ethics Committee of the Institute of Psychiatry of the Federal University of Rio de Janeiro (Opinion: 1.217.635), after meeting the requirements of Resolution nº 466/2012 of the National Health Council. 

## Results 

The mean age of the participants was 40 years (± 12.44), and 61% were male. The most used drug was alcohol, with 55.5%, and 72.2% were dependent on multiple drugs, including marijuana and/or cocaine besides alcohol. 

**Figure 1:**
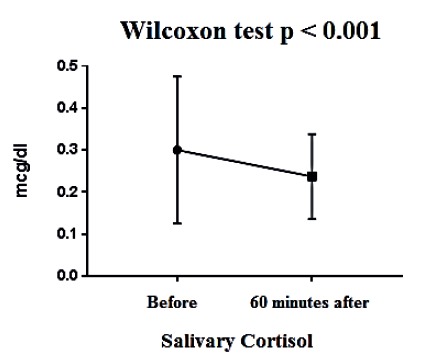
Mean salivary cortisol levels before and 60 minutes after the music therapy intervention. Rio de Janeiro - RJ, Brazil, 2016

**Figure 2:**
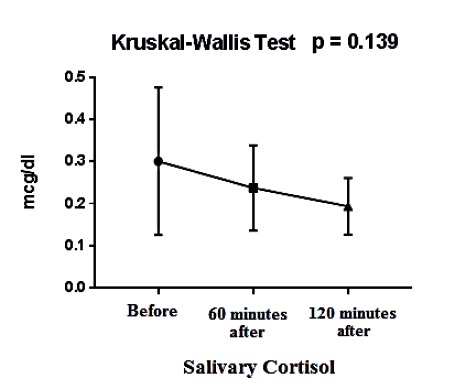
Mean salivary cortisol levels before, 60 minutes after, and 120 minutes after the music therapy intervention. Rio de Janeiro - RJ, Brazil, 2016

After 60 minutes of the music therapy intervention, there was a statistically significant reduction in the mean salivary cortisol levels (*p* < 0.001). A reduction was also noted after 120 minutes, but without statistical significance (*p* = 0.139) (Table 1, Figure 1, Figure 2).

**Table 1:** Means and standard deviations of salivary cortisol levels before, 60 minutes after, and 120 minutes after the music therapy intervention (N = 18). Rio de Janeiro, RJ, Brazil, 2016

Cortisol levels	Mean	Standard deviation	p-value
Before	0.30	0.17	-
60 minutes after	0.23	0.10	<0.001*
120 minutes after	0.19	0.06	0,139^†^

*Wilcoxon test; †Kruskal-Wallis test

## Discussion 

The results of the present study showed that, after 60 minutes of music therapy intervention, there was a statistically significant reduction in the mean levels of salivary cortisol, a biochemical marker of stress. Since stress is a known damaging factor for rehabilitation of chemically dependent people, salivary cortisol is a promising tool to assess the response to neurobiological stress in this population^13^. 

Studies have demonstrated that participants who drop out from treatment for chemical dependence have increased cortisol release and higher peak stress than those who continued under treatment. Each increase in one unit in the peak serum cortisol was related to a four-fold increase in the probability of treatment withdrawal, and other studies have reported a significant increase in cortisol levels in response to stress; only one study presented moderate increase^14^ ^-^ ^15^. 

Although music therapy has been used by complementary medicine in the treatment of various diseases, there are still few studies evaluating the effects of music therapy on chemical dependence. Furthermore, the existing studies made a subjective evaluation of stress, seeking alteration in the perception of the subjects^16^. The present research has the additional positive aspect of precisely showing, in an objective and clear manner, that the stress in chemical dependents can be evaluated by measurement of salivary cortisol, thus revealing a clinical perspective to be used by nurses and other health professionals who provide care of these patients. 

Patterns of drug use can be modified by developmental and environmental interventions, but also by intentional initiatives as in the case of psychotherapy^4^. However, treatment for drug addiction has a number of limitations caused by various factors such as the heterogeneity of addicts, diversity of substances consumed, economic costs involved, difficulties with human resources, and difficulties with specialized materials^5^. Critical factors in the abstinence from additive substances are not related to maturation, treatment or even personal adjustment, but rather to the severity of the addiction and the type of curative experience available to the chemically dependent person^5^. 

In the time of drug abstinence, the dependent person may present irritability, anxiety, emotional stress, sleep disorders, dysphoria, aggressive behaviors and craving, associated with neuroadaptive changes of stress and in the brain reward circuits. Although the occurrence of stress and stressor events is not predictive of relapse, stress reduction alone or combined with pharmacotherapy may be beneficial to lessen craving and maintaining abstinence^17^. 

The use of music in psychiatric patients showed that this resource has great potential to act on the patients’ emotions and behaviors^12^. It is not intended to affirm that music therapy alone is able to cure chemical dependence, but the results of the present research point out, based on the clinical investigation, a significant impact of this therapy, along with psychiatric and psychological treatment, on reduction of stress levels experienced by the chemically dependent individuals, being able to help them in the moments of craving during the abstinence. 

A statistically significant reduction in the mean salivary cortisol was observed 60 minutes after a single session of music therapy, evidencing that this complementary therapy is effective to reduce stress during treatment of chemical dependence.

Around three centuries ago, science began to investigate the effects of music and acoustic vibration in general on the physiological functions of the human being, including the heart and respiratory frequencies. Then, the relation between music and human physiological and psychological responses also began to be investigated^18^. In the nineteenth century, music started to be used by psychiatric institutions, and since then it has been observed that listening to soft melodies effectively calms down agitated patients^18^. 

Some authors affirm that calm songs with little rhythmic variation can be considered sedative because they are able to reduce stress and promote relaxation, indicating their positive usefulness as moderator of therapeutic process^19^. 

In the music therapy performed in this study, which can be defined as interactive, the music therapist and the patients were actively involved in the process of making music^18^. Authors point out that music therapy is not intended to solve the problems faced by patients, but to increase their perception of the available psychosocial resources and strengthen them^19^ ^-^ ^20^. 

When addicts hear or sing songs they chose, there is an important issue of empowerment. Many drug addicts report suffering from low self-esteem; choosing music can show them that they are able to control their choices in their lives. The music therapist usually notices an intense reflection on the words being sung when many participants in the music therapy session express feelings and emotions during the songs, through tears or smiles. 

A Brazilian study showed that a music therapy program was effective in reducing 60% of the level of stress in healthcare professionals working in hospital settings^21^. In turn, a study in Italy found that music therapy is able to reduce stress and the response to stress by showing that plasma cortisol levels had declined in patients who listened to music^22^. Recently, a study conducted to determine the effects of music therapy on self-efficacy of drug avoidance showed that participants undergoing music therapy tended to have the highest self-efficacy scores in drug avoidance^23^. 

Other studies show that musical intervention may be a therapeutic resource that has been increasingly used as complementary therapy to promote relaxation, emotional comfort, and sense of well-being^24^ ^-^ ^25^. In the area of ​​mental health, in particular, music therapists can work with patients who present chemical dependence, treating stress through music therapy techniques^1^. 

The most used chemical substance in the present study was alcohol, with 55.5% of the total. In turn, 72.2% of the individuals were dependent on multiple drugs; besides alcohol, they were also dependent on marijuana and/or cocaine. These results are in line with those of the Brazilian Center for Information on Psychotropic Drugs, which indicates that alcohol is the drug that causes the highest dependence rates^4^. 

For cultural and historical determining reasons, alcohol is the drug with greater availability among the peoples. Drugs, especially alcohol, are intrinsic to our culture and are consumed for various reasons. Alcohol, however, is the substance that causes the greatest number of people to seek specialized treatment. However, with the growth of drug trafficking and the greater variability of substances offered, this may change in the coming years^26^. 

The new and important aspects of the study are the use of music therapy in the daily care of people with chemical dependence. This method has the advantage of being non-invasive, and can be understood as a light healthcare technology, with important benefits for reduction of stress before the possibility of relapse during the course of treatment of chemically dependent patients. 

Music therapy, seen as a light technology, uses attributes of human relationships, essential for bonding in the care space, which was the therapeutic setting in this research. Music can also be regarded as “an innovative technology of care if it is organized as an activity that is at the same time systematic and creative, because it facilitates the expression of emotions, interpersonal communication and because of the possibility of therapeutic effect”^27^. 

It should be mentioned that this study has limitations, one of them being the convenience and small sample, which prevented the analysis of the effect of the therapy on the stress of chemically dependent people in view of the influence of other variables such as sex, age and type of drug to which the patient was addicted, as well as prevented the analysis of the potential effect of alcohol and/or other psychoactive substances on stress reduction. Thus, future studies are recommended to include a control group in order to evaluate the effect of music therapy on stress throughout the treatment of chemical dependence, as well as the possible modification of the effect in the presence of other variables.

## Conclusion

Despite its limitations, this study provided evidence that a single 60-minute session of group music therapy showed to be able to reduce the stress (salivary cortisol levels) of chemically dependent people.
